# Prevalence and Characteristics of Patients with Pressure Ulcers at a Tertiary Hospital in the Eastern Cape, South Africa

**DOI:** 10.3390/healthcare14030293

**Published:** 2026-01-23

**Authors:** Emma Muendo Loko, Nongiwe Linette Mhlanga, Wezile Wilson Chitha, Sibusiso Cyprian Nomatshila, Sikhumbuzo Advisor Mabunda

**Affiliations:** 1 School of Public Health, Walter Sisulu University, Mthatha 5117, South Africa; 2 School of Population Health, University of New South Wales, Sydney 2052, Australia; 3 The George Institute of Global Health, University of New South Wales, Sydney 2052, Australia; 4 The Global Centre for Human Resources for Health Intelligence, Walter Sisulu University, Mthatha 5117, South Africa

**Keywords:** pressure ulcer, Eastern Cape, hospital-acquired pressure ulcer, prevalence of pressure ulcers

## Abstract

**Background/Objectives**: Pressure ulcers in hospitals reflect the nature of care provided. This study sought to describe the prevalence of pressure ulcers and patient characteristics at a large tertiary hospital in South Africa. **Methods**: A descriptive retrospective record review was conducted, and all records of patients with pressure ulcers were included from 1 August 2019 to 31 July 2020. A data abstraction instrument was used to collect data on sociodemographic characteristics, clinical conditions, and pressure ulcer characteristics. Data were analysed using descriptive statistics using the Statistical Package for Social Sciences version 26. **Results**: A total of 12,777 patients were admitted, and 85 records were of adults aged 15 years and above with pressure ulcers. The prevalence of pressure ulcers was 0.7%; of these, 42.4% were hospital-acquired pressure ulcers, while 57.6% had a pressure ulcer on admission. The age group most affected by pressure ulcers was 30–49 years. Most (68.2%) patients had a pressure ulcer on the sacrum, and the majority (34.1%) were admitted to surgical units. Patients who had a hospital-acquired pressure ulcer were three times more likely to be deceased than those who had a pressure ulcer on admission. **Conclusions**: The prevalence of pressure ulcers was lower compared to global and regional prevalences and prevention measures should continue to be implemented at the tertiary hospital.

## 1. Introduction

Pressure ulcers remain a significant healthcare problem, associated with decreased quality of life, pain, disability, and increased hospital stays and expenditures [1]. Pressure ulcers, also known as pressure injuries, pressure sores, or decubitus ulcers [2], are defined as local damage to the skin and underlying tissue resulting from pressure, shearing forces, or both [2,3]. Pressure ulcers developing in the hospital setting are also referred to as hospital-acquired pressure ulcers/injuries [4]. Their occurrence is preventable, and updated guidelines are available to manage them [5]. Globally, the prevalence of pressure ulcers increased from 267,846 in 1990 to 481,423 in 2019 [6]. It is estimated that the prevalence in African hospitals ranges from 3.4 to 18.4% [7]. Though there are estimates from Africa, there remains an underrepresentation of recent studies from the Eastern Cape province in South Africa. Therefore, this study aimed to describe the prevalence of pressure ulcers and the characteristics of patients presenting with them in the Eastern Cape province, South Africa.

Several factors affect the predisposition to pressure ulcer development. These include patient-related factors, such as older age, underweight, the presence of comorbidities, clinical conditions, or spinal cord injuries as well as admission to Intensive Care Units (ICU) [8,9]. There are also healthcare worker-related issues which contribute to the occurrence of pressure ulcers. These include inadequate knowledge, attitudes, and availability of hours for patient care [10,11]. Fidelity in implementing these guidelines may be affected in some cases, with one study noting that repositioning patients is often affected by high workload, a lack of staff, and perceptions of the patients’ haemodynamic instability [12]. In Slovakia, one study found inadequate knowledge (45.5%) and attitudes (67.9%) towards measures of preventing pressure ulcers [10]. A ten-year prevalence study in Sweden found that the provision of pressure-reducing mattresses increased from 74.4% in 2011 to 96.2% in 2020, with a corresponding significant reduction in the prevalence of pressure ulcers from 17.0% to 13.9% [13]. As such, the knowledge of pressure ulcer prevalence may influence healthcare workers’ attitudes and practices in implementing pressure ulcer management interventions.

In South Africa, patient profiles, including chronic disease patterns, differ from those in developed countries like Sweden, which may also influence the predisposition to pressure ulcers. The occurrence of pressure ulcers in South Africa could be affected by the increase in the number of people with chronic conditions characterised by a rising non-communicable disease (NCD) burden. These NCDs are medical conditions characterised by slow progression and long duration, including diabetes and hypertension, among others [14]. Illustrating the increase in NCDs in South Africa, the prevalence of diabetes increased from 3.86% in 2003 to 4.46% in 2016 [15], while hypertension prevalence increased from 38.4% in 2012 to 48.2% in 2016 [16]. The NCD burden occurs in a context with the highest burden of Human Immunodeficiency Virus (HIV). It is estimated that, by the end of 2022, at least 12.7% of people in South Africa were living with HIV [17]. Consequently, this chronic disease burden (including HIV) poses a unique predisposition to pressure ulcer development, as the presence of comorbidities increases the risk of pressure ulcer development [9]. For example, in one tertiary facility, 31.1% of HIV patients presenting at an emergency department were admitted for longer than seven days, with 16.6% requiring ICU admission [18]. Notably, a longer hospital stay and ICU admission are also associated with an increased risk of developing pressure ulcers [9]. Although there is a high chronic disease burden in South Africa, there is also a high prevalence of obesity, with an estimated 25.3% and 32.1% of overweight and obese people, respectively [19]. This obesity may also protect them from pressure ulcer development [9]. Considering a high HIV and NCD burden in South Africa, which may result in long hospitalisation, predisposing patients to pressure ulcers, few recent studies from South Africa have described the pressure ulcer prevalence and the characteristics of patients with pressure ulcers. A previous study was conducted in the urban Western Cape province among patients with spinal cord injuries, and the result was 29.8% [20]. However, evidence from a tertiary hospital serving a predominantly rural population is lacking. Therefore, this study aimed to describe the prevalence and characteristics of patients with pressure ulcers at a tertiary hospital in the Eastern Cape province in South Africa.

## 2. Materials and Methods

The study employed a quantitative approach and a descriptive, retrospective record review study design. A retrospective record review uses prerecorded patient data to answer research questions [21]. In this study, we sought to answer the following questions: (1) What is the prevalence of pressure ulcers at a tertiary hospital in the Eastern Cape province? (2) What are the characteristics of patients presenting with pressure ulcers at the tertiary hospital? To determine prevalence, the record review was conducted using the clinical register of patients admitted between 1 August 2019 and 31 July 2020 in five units: surgical, orthopaedics, high care, medical, and ICU wards. To describe the characteristics of patients with pressure ulcers, data were extracted from the clinical register and information collected from their medical records.

### 2.1. Study Setting

The tertiary hospital is one of ten tertiary facilities in South Africa and the only one located in a predominantly rural province. The hospital is situated in the Eastern Cape province, within the OR Tambo District Municipality. The OR Tambo district had an average of 80% of households living below the poverty line between 2008 and 2017 [22].

### 2.2. Population and Sampling

We reviewed the clinical register of all adult patients. The tertiary hospital admits patients aged more than 12 years to adult units, while those aged 12 and below are admitted to paediatric units. To describe the characteristics of pressure ulcers, we included all patients with pressure ulcers, identified from the clinical register and collected their medical records. The ages of the identified patients in the register ranged from 15 to 93 years.

### 2.3. Data Collection

Data were extracted using a Microsoft Excel instrument developed by the researchers. The extracted data included patient demographic characteristics, such as gender, age, race, marital status, employment status, and the admission ward. Characteristics of the pressure ulcers were also extracted, including whether they were hospital-acquired pressure ulcers or pressure ulcers present on admission, the number of pressure ulcers, and the stage and location of the pressure ulcers. Clinical characteristics included admission diagnosis, duration of hospital stay, comorbidities, presence of faecal and urinary incontinence, and mobility.

### 2.4. Data Analysis

Data were analysed using the Statistical Package for the Social Sciences version (SPSS) 26. Continuous variables were explored for normality using the Shapiro–Wilk test and are summarised using mean, standard deviation, and range if normally distributed, or the median and interquartile range (IQR) if not normally distributed. Categorical variables are summarised using percentages and tables. Contingency tables (crosstabulations) were used to compare differences between hospital-acquired pressure ulcers and pressure ulcers present on admission by the number of pressure ulcers, gender, age, admission ward, condition on discharge, and pressure ulcer stage.

### 2.5. Ethical Considerations

Ethical approval was obtained from the Walter Sisulu University Human Research and Bioethics Committee (Reference: 141/2018), and access approval was obtained from the Eastern Cape Provincial Health Research Ethics Committee (Reference: EC_201904_026). The tertiary hospital also gave permission to conduct the study.

## 3. Results

### 3.1. Prevalence and Characteristics of the Pressure Ulcers

A total of 12,770 adult patients were admitted during the study period. From this population, 85 patients had pressure ulcers. Therefore, the prevalence of pressure ulcers was 0.7%. Out of cases with pressure ulcers, 57.6% had hospital-acquired pressure ulcers, while 42.4% had a pressure ulcer present on admission. As such, the incidence of hospital-acquired pressure ulcers was 0.4%.

The majority (80.0%) had a single pressure ulcer; 12.9% had two; and 3.5% had three, totalling 99 pressure ulcers. Among patients with more than one pressure ulcer, the one with the highest stage was reported, and most (33.3%) had a stage 4 pressure ulcer. The sacrum was the most affected area by pressure ulcers, with 68.2% of patients presenting with a pressure ulcer on the sacrum. Table 1 illustrates the characteristics, sources, and locations of pressure ulcers.

### 3.2. Patients’ Sociodemographic Characteristics

Of the 85 patients with pressure ulcers in the study, 44 (51.8%) were male. The median age was 45 years (interquartile range, IQR = 31–67 years). Concerning the marital status, most patients (51.8%) were single. The patients comprised 88.2% who were unemployed and 68.2% who did not have a reliable source of income. Most (34.1%) patients were admitted to surgical units. The demographic characteristics are summarised in Table 2.

#### 3.2.1. Clinical Characteristics of Patients with Pressure Ulcers

Most (53.0%) patients were admitted for a single diagnosis, with the number of diagnoses on admission ranging from 1 to 5, and the mean number of diagnoses on admission was 1.85 (SD ± 1.07). The most (15.3%) common reason for admission was sepsis. Notably, 5.9% of patients were admitted for pressure ulcers. Table 3 presents the patients’ admission diagnoses.

#### 3.2.2. Patients’ Comorbidities

Patients presenting with pressure ulcers also had comorbidities, with most (49.4%) having one comorbidity. Prevalent patient comorbidities included hypertension (30.6%), HIV (29.4%), diabetes (23.5%), and 11.8% each for cancers and paraplegia. Table 4 below shows patients’ comorbidities.

The median duration of admission was 21 days (range 1–256 days). More than a third (37.6%) of the patients died before discharge, while 62.4% were alive at discharge. Regarding their clinical conditions on admission, most patients (57.7%) were bedridden, 54.1% had urinary incontinence, and 89.4% had a urinary catheter in situ. A total of 68.2% also had faecal incontinence, and 89.4% used a diaper. Table 5 summarises the patients’ clinical conditions.

#### 3.2.3. Crosstabulations

Regarding the number of pressure ulcers, more patients (47.6%) had one hospital-acquired pressure ulcer than those with one pressure ulcer on admission (35.4%). Across all age groups, the highest percentage (17.6%) of patients with a hospital-acquired pressure ulcer were those aged 30–49 years. This was compared with 12.9% among the 30–49-year-old age group who had a pressure ulcer on admission. Across all admission wards, only the high care unit had more patients with pressure ulcers on admission than with hospital-acquired pressure ulcers. For example, more patients (14.1%) admitted to the ICU had a hospital-acquired pressure ulcer than those in the same unit who had a pressure ulcer on admission (2.4%).

Regarding gender, males who had a hospital-acquired pressure ulcer were more compared to those who had a pressure ulcer on admission. Out of all the patients, 30.6% of those who had a hospital-acquired pressure ulcer were discharged alive, while 27.1% of patients with hospital-acquired pressure ulcers were deceased. On the other hand, 32.9% of patients with a pressure ulcer on admission were discharged alive, and 9.4% of patients with a pressure ulcer on admission were deceased. This meant that patients with a hospital-acquired pressure ulcer were about three times more likely to be deceased compared to those with a pressure ulcer on admission (OR = 3.096, 95% CI range 1.2–8.1). Regarding the stage of pressure ulcers, 18.8% of all patients had a stage 4 pressure ulcer on admission, compared with 11.8% who developed a stage 4 hospital-acquired pressure ulcer. Table 6 shows the contingency tables.

## 4. Discussion

The study aimed to describe the prevalence of pressure ulcers among patients admitted to a tertiary hospital in the Eastern Cape province, in South Africa. Findings revealed a prevalence of 0.7% and an incidence of 0.4% for hospital-acquired pressure ulcers. Most patients with pressure ulcers had a single pressure ulcer, and the majority had a stage 4 pressure ulcer. Pressure ulcers were commonly located on the sacrum. The majority of patients included were male, and the age group between 30 and 49 years had the highest representation. This high representation of people aged between 30 and 49 years contrasts with other study findings, which found that elderly patients are more prone to pressure ulcers [8,9]. The study also found that the largest number of patients with pressure ulcers were admitted to a surgical unit, and the common diagnosis was sepsis. The highest number of patients presented with a single comorbidity, with the majority having hypertension, followed by HIV. Most (57.7%) patients were bedridden, with faecal and urinary incontinence. Patients were admitted for a median duration of 21 days.

The high number of patients with a comorbidity also reflects the high HIV and NCD burden in South Africa [15,16,17]. In this study, we found that most patients who had a pressure ulcer had hypertension, followed by HIV, then diabetes. Generally, previous studies have concluded that the presence of a comorbidity is predisposed to pressure ulcers [9]. In this regard, in the South African context, a previous study has also found that the HIV infection is associated with longer hospitalisation, with the need for ICU admission in 16.6% of people living with HIV [18]. Notably, ICU admission is also associated with an increased likelihood of developing pressure ulcers [8,9].

A total of 85 patients comprised the population with a pressure ulcer during the study period. This sample compares with a similar study from Norway [23], which also used data from a single nursing facility and included 73 respondents. In contrast, our sample was small compared to a similar study in the USA, which also used a retrospective record review from a single facility and found 2340 records of pressure ulcers over a one-year period [24]. However, the study by Kirkland-Kyhn et al. (2019) in the USA included 477 due to incomplete records [24], which also characterised our findings. Therefore, our population may be small, limiting its applicability. Although the sample size may be smaller than that of the study by Kirkland-Kyhn et al. (2019), it is worth noting that facility size may vary and influence population size [24].

Regarding the prevalence of pressure ulcers, our study reports a lower prevalence than that reported in the rest of Africa by Anthony et al. (2021), which ranged from 3.4% to 18.4% [7]. Compared with the global prevalence, our findings were also lower than those reported by Li et al. (2020) [25], who found that the prevalence among hospitalised adults was 12.8%, and 8.4% had hospital-acquired pressure ulcers [25]. In South Africa, a study by Joseph and Nilsson Wikmar (2016) [20] found that, between 15 September 2013 and 14 September 2014, the prevalence of pressure ulcers was 29.8% (29/71). This study may not be directly comparable to the findings of Joseph and Nilsson Wikmar, as they included only patients with spinal injuries, whereas we included patients from various units, including surgical units. The results of this study are comparable to those of a US study, which found that the prevalence of hospital-acquired pressure ulcers was 0.05% among patients who underwent vascular surgery [26]. Nonetheless, the lower prevalence compared to the systematic reviews by Li et al. (2020) [25] and Anthony et al. (2021) [7] may be due to several reasons. Firstly, the low prevalence may reflect adequate pressure ulcer management at the tertiary facility in South Africa. Secondly, it could be lower due to the underreporting of pressure ulcers at the facility. Pressure ulcers are often underreported, with one study noting that 67.7% of stage 3 and 4 pressure ulcers were reported [27]. This study also used medical records, which are also known to contain missing data [24], including data on pressure ulcers. Therefore, this may also have contributed to the underreporting of pressure ulcers, a limitation of this study. However, given that the global systematic review by Li et al. [25] did not include studies from Africa and the study by Anthony et al. included only one South African study (Joseph and Nilsson Wikmar, 2016 [20]), highlighting the paucity of literature from the South African context; further studies are warranted.

Concerning the location of pressure ulcers, this study found that most were on the sacrum, followed by the gluteus and the heels. Comparably, several studies [25,28,29,30] also found that the most common site for pressure ulcers was the sacrum. However, there are also studies that contrast our findings. For example, among ICU patients, the most common site of medical device-related pressure ulcers was the ear and nose. In this study, only 2.4% of patients presented with a pressure ulcer on the ear. The study also found that most patients with pressure ulcers were from surgical units, followed by medical units, which is consistent with another study conducted in Indonesia [30]. This study also found that patients who had a hospital-acquired pressure ulcer were three times more likely to be deceased compared to those with a pressure ulcer on admission. A study from Czechoslovakia provides insight into this by noting that patients who die in hospital facilities are more likely to have pressure ulcers of a higher category [31]. The authors further state that among patients who die in a hospital facility, pressure ulcers are reported in 24% of patients during the 365 days before death [31]. Notably, the study’s pressure ulcer prevalence of 24% is higher than the global prevalence reported by Li et al. Nonetheless, there is a need to improve pressure ulcer prevention measures among the dying patients.

### Limitations

Although efforts were made to ensure that the study’s findings were accurate, the study has some limitations. Firstly, the number of patient records included was small. Secondly, some patient information was missing from medical records, which may have affected the interpretation of study findings or contributed to the low prevalence of pressure ulcers.

## 5. Conclusions

Pressure ulcers significantly impact patients’ quality of life, and their occurrence often indicates the quality of healthcare services. Whilst this study found a relatively low prevalence of pressure ulcers compared to the rest of Africa and the world, there is a need for additional studies in South Africa and the continued implementation of measures to prevent pressure ulcers.

## Figures and Tables

**Table 1:** Characteristics of the pressure ulcers (*n* = 85).

Variable	Frequency	Percentage (%)
Source of pressure ulcers		
Hospital-acquired pressure ulcer	49	57.6
Pressure ulcer on admission	36	42.4
Total	85	100.0
Stage of the pressure ulcer		
Stage 1	10	11.8
Stage 2	19	22.4
Stage 3	23	27.1
Stage 4	26	30.6
Unstaged	7	8.2
Number of pressure ulcers		
1	68	80.0
2	11	12.9
3	3	3.5
Missing	3	3.5
Location of pressure ulcers		
Sacrum	58	68.2
Gluteus	15	17.6
Heels	14	16.5
Ear	2	2.4
Coccyx	1	1.2
Hips	5	5.9
Malleolar	2	2.4
Elbow	1	1.2
Trochanter	1	1.2

**Table 2:** Sample demographic characteristics.

Sample Characteristics	Frequency	Percentage (%)
Gender		
Male	44	51.8
Female	41	48.2
Age in years		
15–29	21	24.7
30–49	26	30.6
50–69	22	25.9
70–93	16	18.8
Marital status		
Single	44	51.8
Married	24	28.2
Widow	10	11.8
Not stated	7	8.2
Employment status		
Employed	10	11.8
Unemployed	75	88.2
Source of income		
Employment	10	11.8
Old age grant	11	12.9
Disability grant	1	1.2
None	58	68.2
Not stated	5	5.9
Admission ward		
Surgical	29	34.1
Medical	22	25.9
Orthopaedics	7	8.2
Trauma	4	4.7
High care	9	10.6
ICU *	14	16.5

* ICU—Intensive care unit.

**Table 3:** Patients’ admission diagnosis and number of conditions on admission.

Variable	Frequency	Percentage (%)
Number of diagnoses on admission		
1	45	53.0
2	19	22.4
3	12	14.1
4	8	9.4
5	1	1.2
Admission diagnoses		
Sepsis	13	15.3
Femur fracture	10	11.8
Wet foot gangrene	10	11.8
Dry foot gangrene	2	2.4
Spinal injury	7	8.2
Acute kidney injury	7	8.2
Pneumonia	6	7.1
Traumatic brain injury	5	5.9
Meningitis	5	5.9
Pressure Ulcers	5	5.9
Metabolic acidosis	5	5.9
Acute renal failure	5	5.9
Rib fractures	5	5.9
Uraemic encephalopathy	4	4.7
Subdural haematoma	3	3.5
Polytrauma	3	3.5
Chronic kidney disease	2	2.4
New onset seizures	2	2.4
Status epilepticus	2	2.4
Lung contusion	2	2.4
Acute abdomen	2	2.4
Appendicitis	2	2.4
Perforated peptic ulcer	2	2.4
Pelvic fracture	2	2.4
Hepatocellular carcinoma	2	2.4
Deep vein thrombosis	2	2.4
Leg bone fracture	2	2.4
Diabetic Keto-acidosis	2	2.4
Anaemia or pancytopenia	2	2.4
Dehydration	2	2.4
Other conditions *	32	37.6

* Other conditions refers to all with a frequency of 1 (1.2%), namely, Neurocysticercosis, Spastic paraparesis, Hemiparesis, Degenerative spine disease, Spinal cord compression, Thoraco-lumbar dislocation, Para-spinal mass, Acute Respiratory Distress Syndrome, Pneumothorax, Abdominal Mass, Tuberculosis (TB) abdomen, Blunt Abdominal Trauma, Oesophageal candidiasis, Pleural effusion, Chronic pancreatitis, Severe urinary tract infection, Urethro-cutaneous fistula, Urethro-vaginal fistula, Enterocutaneous Fistula, Acetabulum fracture, Neurogenic bladder, Bilateral Tibia-Fibula fracture, Lower limbs burns, Wound dehiscence, Hypoalbuminaemia, Penile cancer, Gunshot wound, Stabbed abdomen, Chemotherapy complications, Hand necrotising fasciitis, Rheumatoid arthritis, Colon cancer.

**Table 4:** Number and types of comorbidities.

Number of Comorbidities	Frequency	Percentage (%)
0	12	14.3
1	42	49.4
2	18	21.2
3	8	9.4
4	3	3.5
5	1	1.2
Types of comorbidities		
Hypertension	26	30.6
HIV *	25	29.4
Diabetes mellitus	20	23.5
Cancer	10	11.8
Paraplegia	10	11.8
Epilepsy	9	10.6
Chronic kidney disease	5	5.9
Pulmonary tuberculosis	3	3.5
Peripheral vascular disease	2	2.4
Quadriparesis	2	2.4
Other **	7	8.2

* HIV—Human Immunodeficiency Virus. ** Other refers to all with a frequency of 1 (1.2%) comorbidity, and these included Ischaemic heart disease, Rheumatoid arthritis, Tuberculoma, Chronic pancreatitis, Obstructive hydrocephalus, Hydronephrosis, and Deafness.

**Table 5:** Clinical conditions of the patients.

Clinical Condition on Admission	Frequency	Percentage (%)
Mobility		
Yes	5	5.9
Bedridden	49	57.7
Limited	29	34.1
Missing	2	2.4
Urinary incontinence		
Yes	46	54.1
No	37	43.5
Missing	2	2.4
Urinary catheter		
Yes	76	89.4
No	6	7.1
Missing	3	3.5
Diaper		
Yes	76	89.4
No	7	8.2
Missing	2	2.4
Faecal incontinence		
Yes	58	68.2
No	24	28.2
Faecal tube	1	1.2
Missing	2	2.4

**Table 6:** Contingency tables results.

Variables	Pressure Ulcers on Admission Frequency (%)	Hospital-Acquired Pressure Ulcers Frequency (%)	Totals
Number of pressure ulcers			
1	29 (35.4)	39 (47.6)	68 (82.9)
2	5 (6.1)	6 (7.3)	11 (13.4
3	2 (2.4)	1 (1.2)	3 (3.7)
Totals	36 (43.9)	46 (56.1)	82 (100.0)
Age groups in years			
15–29	9 (10.6)	12 (14.1)	21 (24.7)
30–49	11 (12.9)	15 (17.6)	26 (30.6)
50–69	9 (10.6)	13 (15.3)	22 (25.9)
70–93	7 (8.2)	9 (10.6)	16 (18.8)
Totals	36 (42.4)	49 (57.6)	85 (100.0)
Admission unit			
ICU *	2 (2.4)	12 (14.1)	14 (16.5)
Surgical units	14 (16.5)	15 (17.5)	29 (34.1)
High care	6 (7.1)	3 (3.5)	9 (10.6)
Trauma	1 (1.2)	3 (3.5)	4 (4.7)
Orthopaedics	3 (3.5)	4 (4.7)	7 (8.2)
Medical units	10 (11.8)	12 (14.1)	22 (25.9)
Totals	36 (42.4)	49 (57.6)	85 (100)
Gender			
Male	19 (22.4)	25 (29.4)	44 (51.8)
Female	17 (20.0)	24 (28.2)	41 (48.2)
Totals	36 (42.4)	49 (57.6)	85 (100.0)
Condition on discharge			
Alive	28 (32.9)	26 (30.6)	54 (63.5)
Deceased	8 (9.4)	23 (27.1)	31 (36.5)
Totals	36 (42.4)	49 (57.6)	85 (100.0)
Stage of Pressure Ulcer			
Unstaged	3 (3.5)	4 (4.7)	7 (8.2)
1	3 (3.5)	7 (8.2)	10 (11.8)
2	5 (5.9)	14 (16.5)	19 (22.4)
3	9 (10.6)	14 (16.5)	23 (27.1)
4	16 (18.8)	10 (11.8)	26 (30.6)
Totals	36 (42.4)	49 (57.6)	85 (100.0)

* ICU—Intensive Care Unit.

## Data Availability

The data presented in this study are available on request from the corresponding author due to restrictions on confidential patient information.

## Abbreviations

The following abbreviations are used in this manuscript:

ICU	Intensive Care Unit
HIV	Human Immunodeficiency Virus
TB	Tuberculosis
IQR	Interquartile range
SPSS	Statistical Package for Social Sciences
SD	Standard deviation
NCD	Non-communicable disease
